# 

**Published:** 1884-03

**Authors:** 


ORIGINAL COMMUNICATIONS:

Fermentation in the Human Mouth; Its Relations to Caries of the Teeth. W. D. Miller .......... 113
Woman: An Oration Delivered before the American Academy of Dental Science, Boston, Nov. 7, 1883. N. W. Kingsley .......... 119
"Over the Garden Wall." (Is Dental Caries a Disease?) C. M. Wright 135
Iodoform in Dental Surgery. C. F. W. Bodecker .......... 139
Multum in Parvo. J. Smith Dodge .......... 141
An Incident of Practice. C. E. Francis .......... 142

REPORTS OF SOCIETY MEETINGS:

Central Dental Association of Northern New Jersey .......... 143
New Orleans Odontological Society .......... 148
Union Meeting of the Seventh and Eighth District Dental Societies .... 151

EDITORIAL:

Electricity in Dental Practice .......... 154
Advertisements .......... 157
Back Numbers Wanted .......... 157
Left Over .......... 157
Our Book Table .......... 158

CURRENT NEWS AND OPINION:

Michigan State Dental Society .......... 163
Vulcanizing India Rubber .......... 163
State of the Gums in Pregnancy .......... 164
"Can't Afford It." .......... 164
Mississippi Valley Dental Society .......... 165
He Knew It .......... 165
Change of Location .......... 165
Complimentary .......... 166
Dental College in France .......... 166
Texas Dental Association .......... 166
New Use for Electricity .......... 166
Anæsthetics from a Medico-Legal Point of View .......... 167
A Correction .......... 167
Odontological Society of Great Britain .......... 167

